# Front-Surface Potential of Platinized *p*‑InP Photocathodes Probed by Dual-Working-Electrode Measurements

**DOI:** 10.1021/acsenergylett.5c04153

**Published:** 2026-01-19

**Authors:** Weilai Yu, Nathan S. Lewis

**Affiliations:** † Division of Chemistry and Chemical Engineering, 127-72 Noyes Laboratory, 6469 California Institute of Technology, Pasadena, California 91125, United States; ‡ Department of Chemical Engineering and Applied Chemistry, 7938 University of Toronto, Ontario ON M5S 3E5, Canada; § Beckman Institute, California Institute of Technology, Pasadena, California 91125, United States

## Abstract

The front-surface potential (*E*_fr_) of *p*-InP/Pt photocathodes performing the hydrogen-evolution reaction has been probed under operating conditions using a dual-working-electrode (DWE) method. The DWE data are consistent with expectations for proposed stability mechanisms for *p*-InP photocathodes. Specifically, *E*_fr_ for Pt-modified *p*-InP adopts a value near the reversible hydrogen electrode (RHE) potential, consistent with kinetic suppression of metallic In^0^ formation. The data provide a rapid, operando metric of interfacial catalyst activity, and indicate that *E*_fr_ for the *p*-InP/Pt junction is governed by surface catalytic kinetics rather than by the applied back-contact potential (*E*_b_).

## Graphical abstract

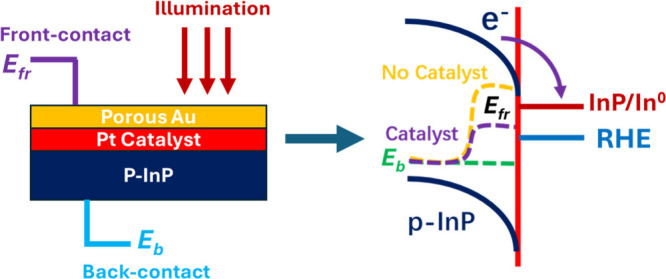

Understanding electrochemical reactions at buried semiconductor–catalyst interfaces is a central challenge for solar fuels, because conventional photoelectrochemical (PEC) metrics—back-contact biasing, chopped-light current density–potential (*J*–*E*) scans, and open-circuit photovoltage—often conflate the energetics of the semiconductor junction with catalytic overpotentials,^1–4^ obscuring the origins of performance and stability limitations in photocathodes,^5–7^ including high-photovoltage *p*-InP systems.^8,9^

Here, we report results of an operando dual-working-electrode (DWE) method in which the back-contact potential (*E*_b_) was independently controlled while the front-surface potential (*E*_fr_) of a catalyst-coated *p*-InP photocathode was monitored directly during hydrogen evolution, providing real-time access to the electrochemical potential at the semiconductor/catalyst/liquid interface under working photoelectrochemical conditions. The Supporting Information describes the fabrication and testing of the DWE (Figure 1a and Figure S1). A thin (∼10 nm) nanoporous Au front contact maintained electrical continuity, served as an effective probe of *E*_fr_, and permitted electrolyte access to the underlying Pt catalyst (Figure 1b).^10,11^ Unlike prior DWE studies that were performed in the dark,^9^ this configuration allowed measurement of the illuminated, operating photocathode, enabling direct measurement of the surface potential that governs interfacial kinetics and electrode degradation.

**Figure 1:**
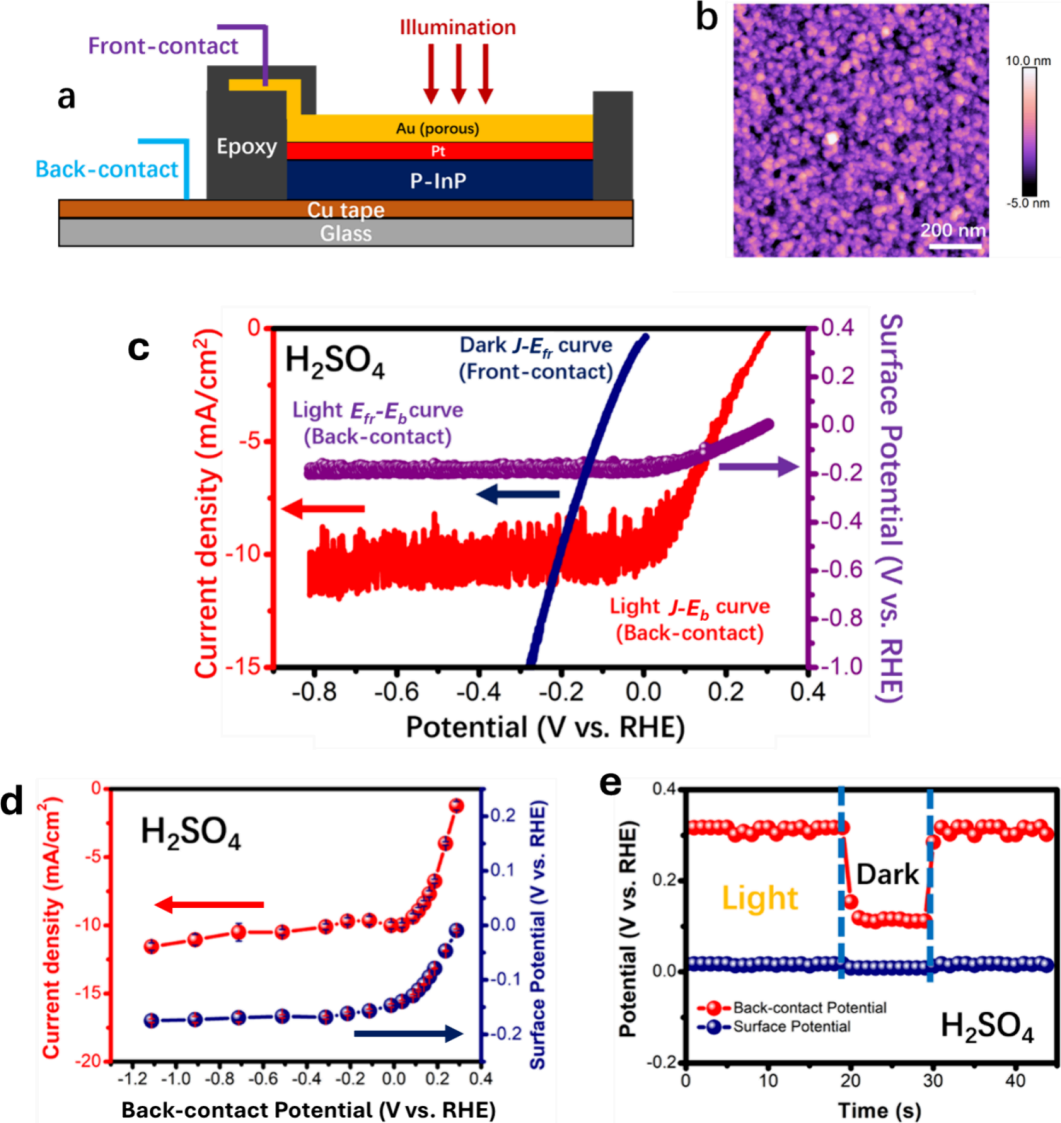
(a) Cross-sectional schematic of an illuminated *p*-InP/3 nm Pt/10 nm Au (*p*-InP/3Pt/10Au) dual-working-electrode (DWE) structure, showing both back and front contacts. (b) Atomic force microscopy (AFM) image of a 10-nm Au thin film deposited by electron-beam (e-beam) evaporation, exhibiting a nanoparticulate and porous morphology (*R*_q_ = 1.9 nm). (c) Comparison of current density vs back-contact potential (*J*–*E*_b_, red) and front-contact vs back-contact potential (*E*_fr_–*E*_b_, purple) curves under 100 mW cm^–2^ of illumination (ELH-type W-halogen bulb), together with the current density vs front-contact potential (*J*–*E*_fr_, blue) curve in the dark, for the DWE in (a), measured in 1.0 M H_2_SO_4_(aq). Figure S2 shows results for the same electrode in 1.0 M KOH­(aq). Scan rate: 50 mV s^–1^. (d) Surface potentials *E*_fr_ (blue) and current densities (red) measured under potentiostatic conditions (held for 60 s) at each applied *E*_b_ in 1.0 M H_2_SO_4_(aq). (e) Comparison of open-circuit potentials at the back and front contacts under dark and illuminated conditions, respectively, in 1.0 M H_2_SO_4_(aq).

We test the hypothesis that, in the light-limited regime, the stability of the photocathode is dictated by the instantaneous *E*_fr_ at the illuminated surface rather than solely by the externally applied bias. Real-time *E*_fr_ measurements cleanly decouple the semiconductor photovoltage from the catalytic overpotential, directly linking the photocurrent to the catalytic driving force.^11,12^ This operando view identifies when corrosion pathways are activated and connects the surface-potential trajectories to long-term stability, extending mechanistic frameworks for *p*-InP photocathodes.^8^

Sweeps of the potential of the back contact (*E*_b_) from open circuit to −0.9 V versus the reversible hydrogen electrode (RHE) (−0.9 V_RHE_) did not shift the surface potential (*E*_fr_) of the *p*-InP/3 nm Pt/10 nm Au DWE. Instead, *E*_fr_ plateaued at −0.17 V_RHE_ in 1.0 M H_2_SO_4_(aq) and at −0.23 V_RHE_ in 1.0 M KOH­(aq), corresponding to light-limited photocurrents densities (*J*_ph_) of −10.5 ± 0.2 and −11.7 ± 0.2 mA cm^–2^, respectively, under 100 mW cm^–2^ of illumination (Figure 1c and Figure S2). Hence, the Pt/liquid interface controlled the “pinned” *E*_fr_ value that was required to sustain the photocurrent in this system.

At open circuit, the measured *E*_fr_ value of *p*-InP/3 nm Pt/10 nm Au DWEs equilibrated to ∼0 V_RHE_, consistent with equilibration of the Pt to the RHE potential (Figure 1d,e). Direct control of *E*_fr_ through the front contact in the dark yielded *J*–*E*_fr_ data that were identical to the polarization behavior of metallic Pt for hydrogen evolution, confirming that the measured *E*_fr_ probes the electrochemical potential at the surface of the Pt HER catalyst (Figure S3). The voltammetric and potentiostatic data were mutually consistent, and demonstrate that *E*_fr_ scaled with current density and surface kinetics rather than with *E*_b_ (Figure 1d,e and Figure S3). These results establish that *E*_fr_ provides a direct, operando method to decouple the semiconductor photovoltage from interfacial catalytic overpotentials.

At comparable values of *J*_ph_, DWE electrodes fabricated with pre-photoelectrodeposited Pt nanoparticles showed *E*_fr_ pinning at −0.12 V_RHE_ in 1.0 M H_2_SO_4_(aq) and at −0.23 V_RHE_ in 1.0 M KOH­(aq) (Figure 2a,b and Table S1). These values are essentially identical to those obtained with sputtered Pt, indicating that *E*_fr_ does not depend on the Pt deposition method. The shift in *E*_fr_ between acidic and alkaline electrolytes arises from the pH-dependent HER overpotential on Pt.

**Figure 2:**
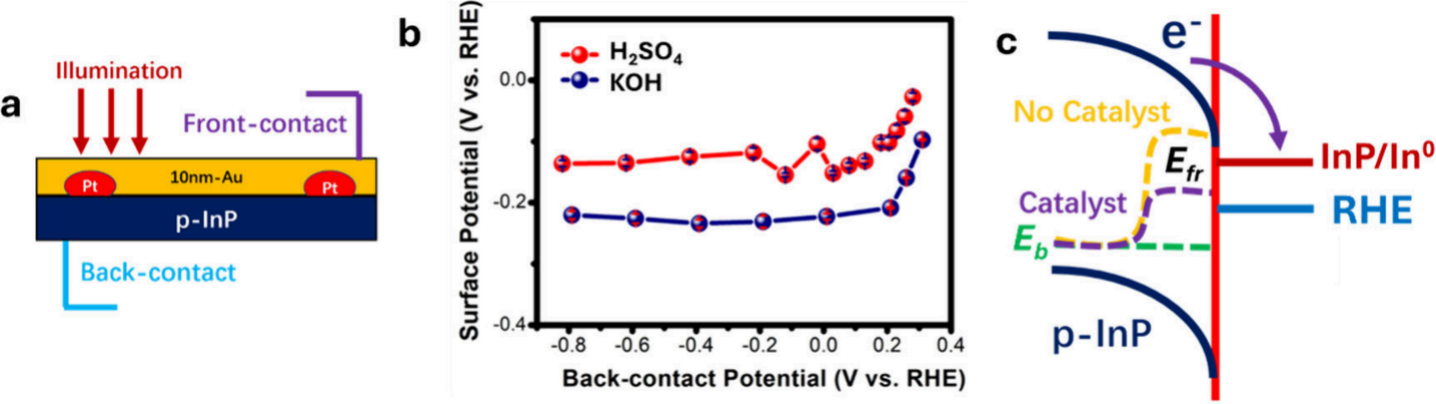
(a) Schematic illustration of a *p*-InP DWE with photoelectrodeposited Pt particles. (b) Comparison of the measured *E*_fr_ of the DWE in (a) as a function of *E*_b_ in 1.0 M H_2_SO_4_(aq) and 1.0 M KOH­(aq), respectively, under 100 mW cm^–2^ of illumination. (c) Kinetic stabilization mechanism of p-InP photocathodes with active HER catalysts (Pt) to suppress cathodic corrosion and prevent the formation of metallic In^0^.

When Pt was replaced with a Au front contact (Figure S4), the *p*-InP/14 nm Au DWE exhibited *E*_fr_ values of −0.30 V_RHE_ in 1.0 M H_2_SO_4_(aq) (*J*_ph_ = −8.1 ± 0.3 mA cm^–2^) and −0.47 V_RHE_ in 1.0 M KOH­(aq) (*J*_ph_ = −7.5 ± 0.3 mA cm^–2^). These potentials are substantially more negative than those measured for *p*-InP/3 nm Pt/10 nm Au photoelectrodes under nominally identical operating conditions, indicating that the porous Au layer alone is a substantially less active HER catalyst than Pt. The more negative front-surface potentials observed with Au are consistent with the higher HER overpotential of Au relative to Pt, and confirm that the pinned value of *E*_fr_ reflects the catalytic overpotential required to sustain the photocurrent. To sustain a given current density, a less active catalyst drives the surface potential to more negative values, showing that photocathode performance is governed by catalyst kinetics and that, in Pt-modified electrodes, the Au overlayer does not dominate the interfacial behavior. For a given photocurrent density, *E*_fr_ is pinned at a catalyst-dependent potential, making the DWE platform a rapid, quantitative benchmark that links kinetics to operating surface potential and enables direct evaluation of catalyst composition, morphology, and contact strategy under working conditions.

Notably, the DWE data are consistent with proposed stability mechanisms for *p*-InP photocathodes (Figure 2c).^8,13,14^ Because the plating potential for In^0^ from InP (approximately −0.31 V_RHE_) is more negative than the RHE potential, the intrinsically high HER overpotential at etched *p*-InP can drive the surface to sufficiently negative potentials to enable cathodic corrosion via metallic In^0^ formation, introducing kinetic competition between In reduction and hydrogen evolution. The conversion of *p*-InP to In^0^ is thermodynamically less favorable than HER, so kinetic stabilization can render *p*-InP quasi-stable, in accord with Gerischer’s classification.^15^ Consistently, decoration of *p*-InP with Pt suppresses the formation of In^0^ by preferentially channeling photogenerated carriers into the HER, yielding more positive operando surface potentials than nonplatinized, etched p-InP photocathodes under comparable applied bias conditions.^8^

Our results directly confirm this picture: for Pt-modified *p*-InP, *E*_fr_ remains near RHE, consistent with kinetic suppression of In^0^ formation, whereas less active catalysts such as Au drive *E*_fr_ to more negative values that favor corrosion. Beyond decoupling photovoltage from catalytic kinetics, operando DWE establishes a mechanistic link between surface overpotential, catalyst activity, and stability, and highlights practical levers to bias selectivity toward the HER—optimizing catalyst loading and morphology, introducing ultrathin interlayers that hinder In^0^ nucleation, and tuning the electrolyte composition and applied bias to keep *E*_fr_ within an HER-favorable window. More broadly, DWE links the surface potential to catalyst kinetics operando, rapidly exposing bottlenecks and degradation pathways to guide the development of scalable, robust photocathodes.

## Supplementary Material



## Notes

Experimental procedures for DWE photocathode fabrication and testing, electrochemical cell configuration and supplementary electrochemical data (Figures S1–S4), and summary table of measured surface potentials and photocurrent densities (Table S1) (PDF)
