# Supplementary material for: Front-Surface Potential of Platinized p‑InP Photocathodes Probed by Dual-Working-Electrode Measurements
Source: ACS Energy Lett. 2026 Jan 19;11(2):1601–4. doi: 10.1021/acsenergylett.5c04153 (PMC12910661; doi:10.1021/acsenergylett.5c04153)
Supplement: Supplementary file 1 [file nz5c04153_si_001.pdf]

Supplementary Information

Front-Surface Potential of Platinized p-InP Photocathodes Probed by Dual-Working-Electrode Measurements

Weilai Yu[1,2], Nathan S. Lewis[1,3*]

[1]Division of Chemistry and Chemical Engineering

127-72 Noyes Laboratory

California Institute of Technology

Pasadena, CA, 91125, United States

[2]Department of Chemical Engineering and Applied Chemistry

University of Toronto

Ontario, ON, M5S 3E5, Canada

[3]Beckman Institute

California Institute of Technology

Pasadena, CA 91125, United States

Corresponding Author: nslewis@caltech.edu

Fabrication and Testing of Dual-working Electrode (DWE) Photocathodes

DWE photocathodes were fabricated by using Ag paint to affix p-type InP wafers (10 nm Zn/90 nm Au back-contact layer with annealing) to Cu coils that were supported by a glass slide, followed by encapsulation of the edges of the InP with epoxy to define the active area of the photoelectrode (**Figure 1a and Figure S1**).[1] Pt was deposited on the p-InP surface via e-beam evaporation or light-assisted electrodeposition. The Pt was then capped with a thin (~10 nm) nanoparticulate Au film that served as the front electrical contact. The Au layer extended along the epoxy boundary to form a mechanically robust pad for the E_{fr} probe, and a second epoxy layer insulated the electrical lead. The e-beam-evaporated Au film exhibited an interconnected nanoparticulate morphology with a root-mean-square roughness (R_{q}) of 1.9 nm (**Figure 1b**). This porous Au structure allowed electrolyte penetration to the underlying Pt layer, enabling efficient hydrogen evolution reaction (HER) activity at the catalyst–electrolyte interface.[2,3]

DWE measurements were conducted in a custom electrochemical cell with a glass window (**Figure S1**), under a continuous H_2 purge and with 100 mW cm^{-2} of illumination (1 sun). This configuration allowed independent control of the back-contact potential (E_{b}) while monitoring the front-surface potential (E_{fr}) either at open circuit or under applied bias, as measured by a separate potential probe of a BioLogic potentiostat. Both $E_b$and E_{fr}were measured against a common reference electrode, either a saturated calomel electrode (in H_2SO_4) or a Hg/HgO electrode (in KOH), allowing conversion to the RHE scale and accurate determination of the difference between $E_b$and E_{fr}. The integrated platform provided direct, real-time insight into the electrochemical potential at the catalyst interface under operating photoelectrochemical conditions.

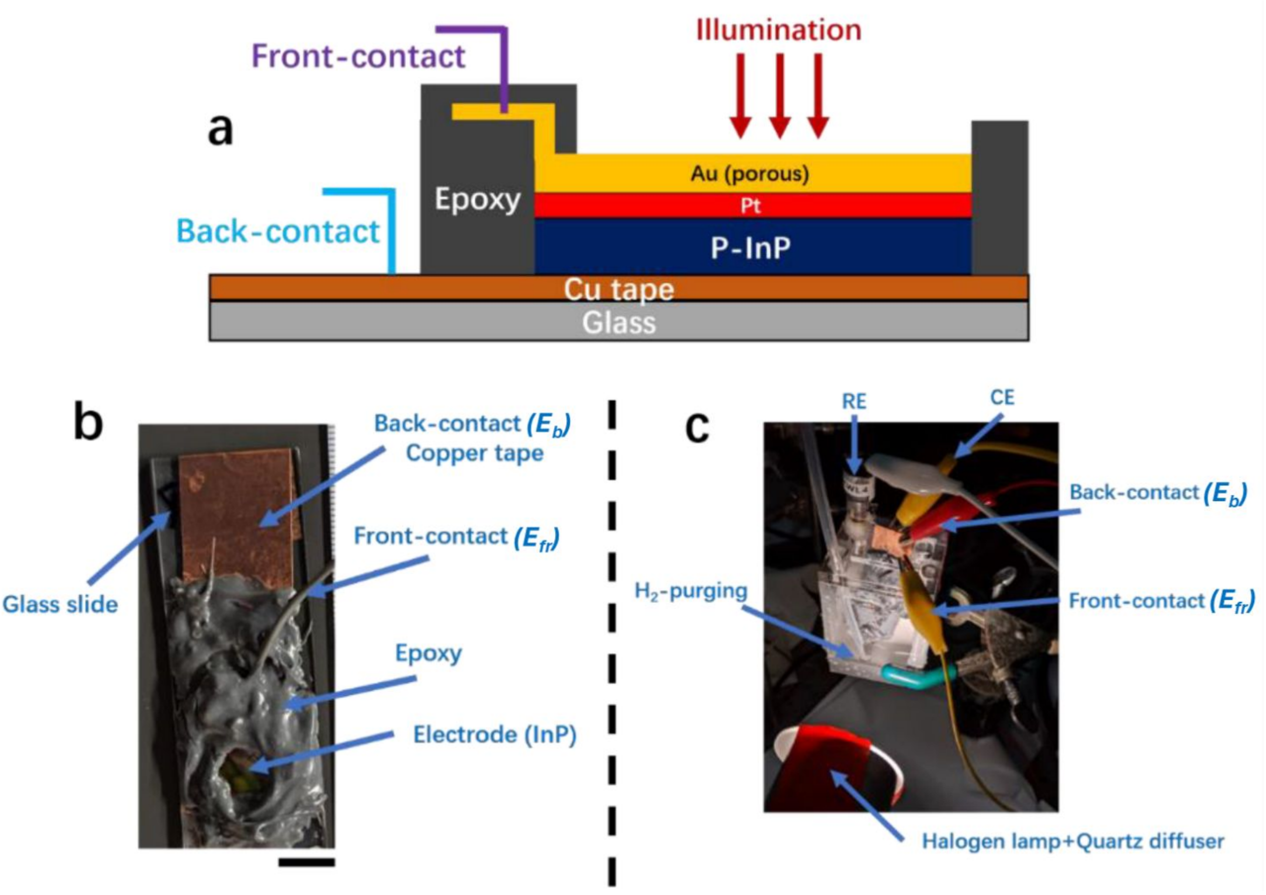

Figure S1. (a) Schematic illustration of a p-InP dual-working-electrode (DWE) structure. (b) Optical image of an as-prepared p-InP/Pt/Au DWE (scale bar: 1 cm). (c) Experimental setup for four-electrode DWE measurements under H_2(g) purging and 100 mW cm^{-2} of illumination using an ELH-type tungsten–halogen lamp.

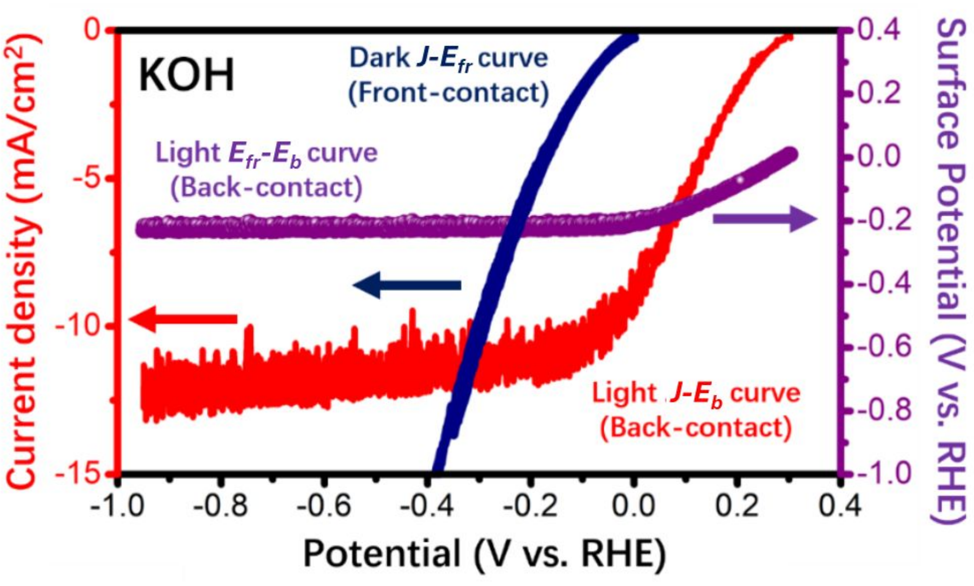

Figure S2. Comparison of the current density vs. back-contact potential (J–E_b, red) and front-contact vs. back-contact potential (E_{fr}–E_b, purple) curves under 100 mW cm^{-2} of illumination, along with the current density vs. front-contact potential (J–E_{fr}, blue) curve measured in the dark, for the same DWE shown in **Figure 1**, but in contact with 1.0 M KOH(aq).

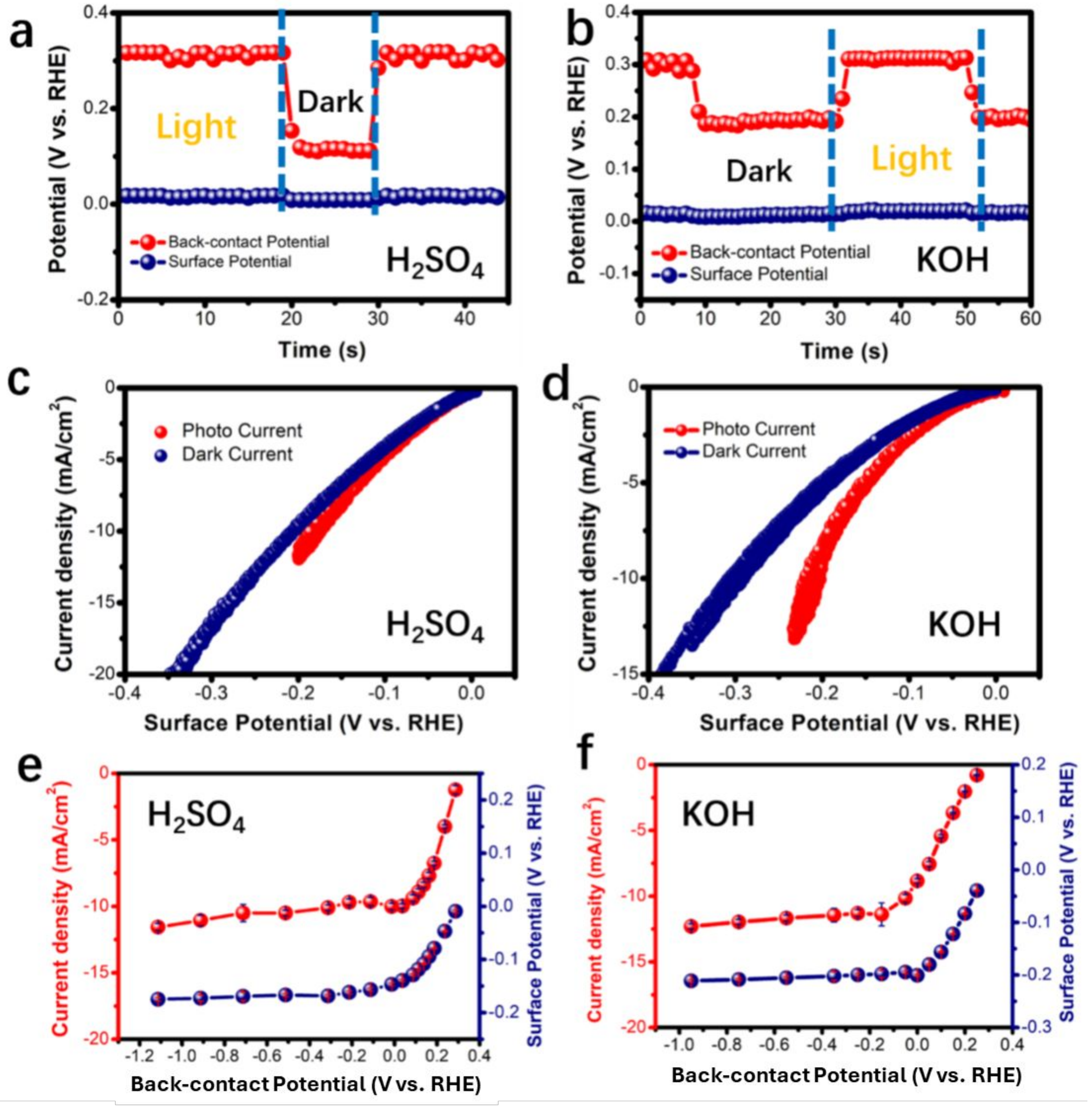

Figure S3. (a–b) Comparison of open-circuit potentials at the back and front contacts under dark and illuminated conditions for the DWE shown in **Figure 1**, measured in (a) 1.0 M H_2SO_4(aq) and (b) 1.0 M KOH(aq). (c–d) Comparison of the measured J–E_{fr} curves obtained by controlling either E_b under illumination (denoted as "photocurrent") or E_{fr} in the dark (denoted as "dark current") in (c) 1.0 M H_2SO_4(aq) and (d) 1.0 M KOH(aq). (e–f) Comparison of measured surface potentials (blue) and current densities (red) under potentiostatic conditions (held for 60 s) at each applied E_b in (e) 1.0 M H_2SO_4(aq) and (f) 1.0 M KOH(aq).

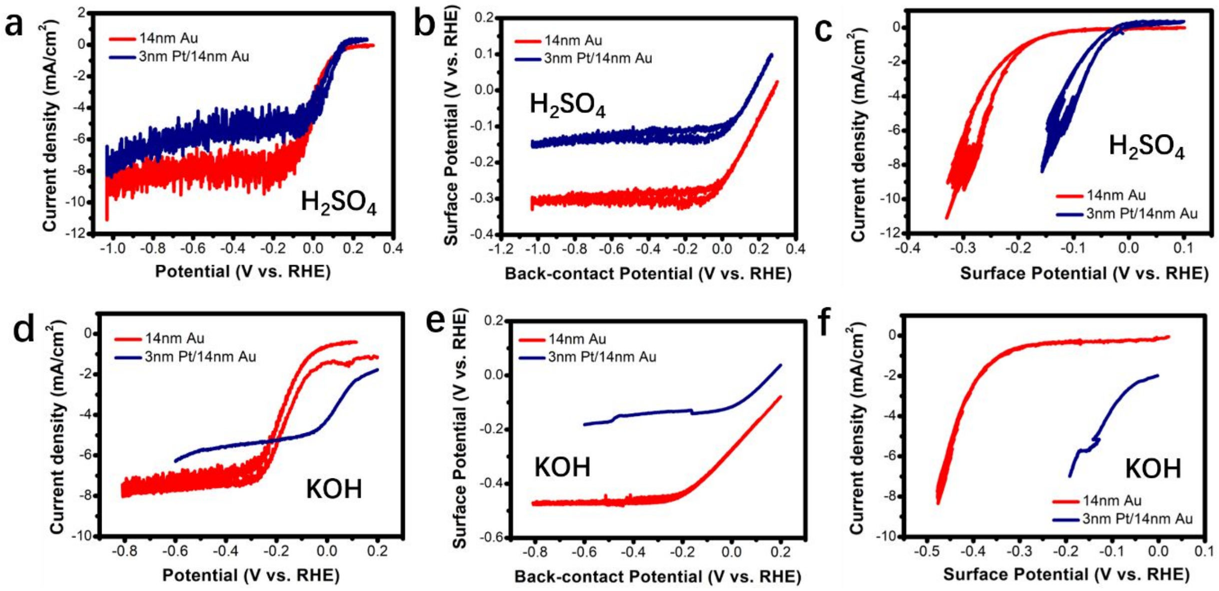

Figure S4. Comparison of (a, d) J–E_b curves, (b, e) E_{fr}–E_b curves, and (c, f) J–E_{fr} curves (E_{fr} at open circuit) for p-InP/3Pt/14Au (blue) and p-InP/14Au (red) DWEs in contact with (a–c) 1.0 M H_2SO_4(aq) and (d–f) 1.0 M KOH(aq) under 100 mW cm^{-2} of illumination. Scan rate: 50 mV s^{-1}.

Table S1. Summary of measured E_{fr} at J_{ph} for different p-InP DWEs.

Electrolyte	Electrode[a]	J_{ph} (mA cm^{-2})[b]	E_{fr} (V vs. RHE)
H_2SO_4	Ed-Pt/10Au[c]	−13.1 ± 0.2	−0.12 ± 0.008
	3Pt/10Au	−10.5 ± 0.2	−0.17 ± 0.003
	14Au	−8.1 ± 0.3	−0.30 ± 0.005
	3Pt/14Au	−5.8 ± 0.3	−0.13 ± 0.003
**KOH**	Ed-Pt/10Au	−13.7 ± 0.2	−0.23 ± 0.006
	3Pt/10Au	−11.7 ± 0.2	−0.23 ± 0.008
	14Au	−7.5 ± 0.3	−0.47 ± 0.003
	3Pt/14Au	−6.0 ± 0.05	−0.17 ± 0.001

[a] Numbers denote the thickness (nm) of evaporated Pt or Au films.

[b] J_{ph} represents the light-limited current density measured at $E_b = -0.4/-0.5$ V vs. RHE.

[c] "Ed" denotes electrodeposited Pt.

References

(1) Boettcher, S. W.; Strandwitz, N. C.; Schierhorn, M.; Lock, N.; Lonergan, M. C.; Stucky, G. D. Tunable Electronic Interfaces between Bulk Semiconductors and Ligand-Stabilized Nanoparticle Assemblies. *Nat Mater* **2007**, *6* (8), 592–596. https://doi.org/10.1038/nmat1943.

(2) Lin, F.; Boettcher, S. W. Adaptive Semiconductor/Electrocatalyst Junctions in Water-Splitting Photoanodes. *Nat Mater* **2014**, *13* (1), 81–86. https://doi.org/10.1038/nmat3811.

(3) Laskowski, F. A. L.; Nellist, M. R.; Venkatkarthick, R.; Boettcher, S. W. Junction Behavior of N-Si Photoanodes Protected by Thin Ni Elucidated from Dual Working Electrode Photoelectrochemistry. *Energy Env. Sci* **2017**, *10* (2), 570–579. https://doi.org/10.1039/C6EE03505A.
